# Replication of influential studies on biomedical, social, behavioural and structural interventions for HIV prevention and treatment

**DOI:** 10.1371/journal.pone.0240159

**Published:** 2020-10-20

**Authors:** Eric W. Djimeu, Anna Heard

**Affiliations:** 1 Global Alliance for Improved Nutrition, Washington, DC, United States of America; 2 CEREG, University of Yaounde II, Yaounde, Cameroon; 3 Innovation for Poverty Action, Washington, DC, United States of America; University of the Witwatersrand, SOUTH AFRICA

## Abstract

Replication is an important tool to promote high quality research and ensure policy makers can rely on studies in making guidelines or funding programs. By ensuring influential studies are replicable we provide assurance that the policies based on these studies are well-founded and the conclusions and recommendations are robust—to different estimation models or different choices. In this paper, we argue that replication is not only useful but necessary to ensure that an author’s choice in how to analyse data is not the only factor that determines whether an intervention is effective or not. We also show that while most research is done well and provides robust results, small differences can lead to different interpretations and these differences need to be acknowledged. This special issue highlights 5 such replication studies, which are replications of influential studies on biomedical, social, behavioural and structural interventions for HIV prevention and treatment. We reflect on their findings. Four out of five studies, which conduct push button replication and pure replication, were able to reproduce the results of the original studies with minor differences, mainly due to minor typographical errors or rounding differences. The analysis of the measurement and estimation analyses conducted in these five studies reveals that the original results are not very robust to alternative analytical approaches, especially when these results rely on a small number of observations. In these cases, the original results are weakened. Furthermore, in contrast to the original papers, two of the five included replication studies conducted a theory of change analysis—to explore how or why the interventions work (or do not) not just whether the intervention works or not. These two analyses indicate that the estimated impacts of the interventions are drawn from few mediators. In addition, they demonstrate that, in some cases, a lack of effect may be related to lack of adequate exposure to the intervention rather than inefficacy of the intervention *per se*. However, overall, the included replication studies show that the results presented in the original papers are trustworthy and robust, especially when based on larger sample sizes. Replication studies can not only verify the results of a study, they can also provide additional insights on the published results, such as how and why an intervention was effective or less effective than expected. They can thus be a tool to inform the research community and/ or policymakers about whether and how interventions could be adopted, which need to be tested further, and which should be discontinued because of their ineffectiveness. Thus, publishing these replication studies in peer-reviewed journals makes the work public and publicized. The work advances knowledge, and publication should be encouraged, as it is for other types of research.

## Introduction

There is a replication crisis—in psychology, but now it is extending beyond, to economics and other social sciences. Recent press around non-replicable psychology studies (see [1]), and the Stanford Prison Experiment [2] suggest that replication of influential studies can provide important verification to build confidence in the integrity of the results. Beyond psychology, Chang and Li [3] of the Federal Reserve published a paper in 2015 suggesting economics research is “usually not” replicable. Another group of researchers led by Camerer reviewed 21 studies in the social sciences published in Nature and Science between 2010 and 2015 and found a significant effect in the same direction as the original study in only 62% of studies, and the average effect was about 50% of the original [4].

Since the initial recognition of HIV over three decades ago, work on HIV prevention and treatment has yielded a series of effective biomedical, social, behavioural and structural interventions [5]. Most successful HIV prevention interventions are tested among specific populations in small-scale settings or controlled trials. Few have been adopted at a population level to prevent HIV [6]. In addition, the scale of the epidemic and an urgent need to provide care and treatment to a quickly growing population of people living with HIV has resulted in some treatment recommendations or treatment practices being based on a single study or only a few studies. Given the questions around validity and replicability of results, the importance of the effort to prevent HIV transmission and achieve epidemic control and the magnitude of the effort needed to scale up any evidence-based intervention require the study results be carefully reviewed, understood and confirmed.

Replication of a study’s results using the same data, which we call internal replication, can help improve the quality and reliability of studies used for policymaking. It does this both by ensuring the study results are reliable and by promoting the use of best practices and transparent research. Replication ensures reliability not just by verifying that the results are not affected by error (e.g. coding or data cleaning errors, typographical errors), but also by assessing whether the results are affected by different analytical choices. In a report in The Conversation [7], Andreas Ortmann notes that the reason some studies are not replicable isn’t fraud, it’s that research is messy and “reasonable people can reasonably disagree on the various calls that have to be made.”

Applied social science empirical research, including health systems research or health intervention research at the population or community level, is not like hard science or biomedical efficacy trials, or even clinical psychology trials done in an office, where validation would more likely come from an external replication—repeating the experiment with a new sample or new group of patients [8]. “Real world” applied social science research relies on statistical methods to adjust for bias, assumptions researchers make to justify their use and the indicators they select or create to measure social and behavioural or economic concepts. These assumptions and choices are human choices, subject to error and differences of opinion. Replication can help assess whether these assumptions and choices are robust to reasonable alternatives. Brown, Cameron and Wood also suggest that replication can address publication and reporting bias by exploring how selective the reported results seem. Still, they note that it cannot uncover studies that were never published [8].

Replication supports transparency and good research and reporting practices by making thorough documentation essential. If study replication becomes routine, authors will need to ensure that they include clear descriptions of their steps in data cleaning and analysis and that data are publicly available.

3ie launched a replication program close to seven years ago to support these aims. 3ie is a grant-making organization that supports the generation and use of high-quality evidence to inform decision-making (www.3ieimpact.org/about-us). They fund, produce, quality assure and synthesize rigorous evidence that examine what works, for whom, why and at what cost in low- and middle-income countries. The replication program has funded 23 replications through four rounds of funding. In addition, 3ie now performs push-button replications of all studies they have funded and reports which studies they have not been able to replicate due to an inability to obtain the necessary data or code.

3ie published many of the supported replication studies in a journal supplement in September 2018 [9] that highlighted the accomplishments of the 3ie initiative. This current supplement includes several new replication studies that focus on HIV prevention and treatment. We believe that publication in a peer reviewed journal, such as PLOS, is critical. Peer review helps ensure that the researchers have used appropriate methods to assess robustness and that they have provided an accurate and appropriate interpretation. Publication in a respected journal also provides citable evidence of the robustness of the original studies. In addition, bundling the studies into a special issue or supplement provides an opportunity to group the studies and emphasize their relevance. PLOS ONE represents an ideal journal for this group of studies, due to its audience and its publication record of HIV-related studies. We hope that this supplement demonstrates the value of replication and provides a greater understanding of internal replication and its different components.

### The selection process of studies included in this supplement and the implementation of replication window 3 (RW3)

The grant opportunity, which 3ie calls a “grant window”, through which the studies included in this supplement were funded, aimed to promote the replication of five influential studies. The researchers were able to identify studies that they felt were influential in HIV prevention and treatment. Other 3ie-funded replication grant windows have provided a list of influential studies from which to choose, but this grant window allowed for greater flexibility in selection of studies to replicate. The goal of the added freedom for researchers to self-select a study was to allow for a wider perspective of what researchers considered influential. Researchers were required to justify their selection. The only restriction 3ie made was that a proposed study needed to be in 3ie’s online Impact Evaluation Repository (IER). The IER is a database of all known qualified impact evaluations of development interventions in low or middle-income countries. To qualify, studies need to employ a randomized or statistical counterfactual, an unbiased comparison group that allows for attribution.

To identify highly influential studies in HIV prevention and treatment, four of the five authors considered the most recent 94 studies available in the 3ie Repository published between 2011–2014 [10–13]. Using the number of citations from the Web of Science and the months since publication, they calculated a citation rate. They weighted each citation rate with the journal Impact Factor to identify the studies with the most impact. From these top studies, each of the four authors selected one that most interested them. The fifth group of authors selected their study to replicate from a 2011 WHO recommendation based on three studies [14]. The recommendation relates to people co-diagnosed with HIV and tuberculosis (TB). TB is the most common presenting illness and leading cause of death among people living with HIV [15]. Based on these three studies, the WHO recommended (and still recommends) earlier initiation of HIV treatment (after initiation of TB treatment) for patients with a very low CD4 count (<50 cells/mm^3^) than those with a higher count. CD4 levels are a measure of immune health. Given the influence of these three studies, the authors decided to replicate one of them—the largest.

Each grantee submitted a replication plan, which is like a pre-analysis plan. Plans were finalized and posted on the 3ie website after revisions based on comments from 3ie and external reviewers. Any deviations from the original plans required explanation in the final publication. Each replication study contains two major components, a pure replication—replicating the original authors’ methods to attempt to replicate the results presented in the original paper, and a measurement and estimation analysis (MEA)—to assess the robustness of the original study’s methods and interpretations. The pure replication can include a push button replication (PBR) that uses the original authors’ statistical codes to replicate the results as well as attempting to replicate the results without the code, based only on the methods described in the paper. The MEA could include, for example, the use of different estimation models, differently created standard errors, heterogeneity or sub-group analyses, or different treatment of missing data. As mentioned earlier, the choice of an estimation model relies on human decisions. In many cases there is not one right answer. The choice of a model can rely more on the background of the researcher and the field to which they belong (e.g. epidemiology versus economics) than the inherent biases associated with different choices. Space limitations can prevent multiple models from being presented. Occasionally, new eyes can see new possibilities. In replication studies, authors report why they believe an alternative specification is helpful and readers are allowed to judge the value of the alternative analysis.

As part of the replication grant program, grantees were assigned an external project advisor (a recognised researcher from academic or a research institution) who, along with 3ie staff, provided reviews at multiple steps of the replication research, including for the replication plan, the pure replication, and the draft final replication study. We also solicited and shared comments from an additional, anonymous, external reviewer at the draft final replication study stage. In addition, grantees were required to adhere to the programme’s notification and communication policy, which set out standard procedures for communication between the replication researchers and the original authors [16]. These procedures, which aim to reduce frictions within the replication process, include sharing the pure replication results with the original authors upon its completion and before the robustness assessment.

### The included replication studies

The five included studies used thoughtful rationale to identify “influential” publications. Each replication study carefully replicated the original authors’ methods and, where possible, statistical code. The measurement and estimation analyses used thoughtful alternative models or assessed new aspects to provide a broader interpretation of the findings (Table 1).

**Table 1:** Presents a summary of findings and methods used in the original paper and the replication study.

Study	Methods used in the original paper	Methods used in MEA of the replication	Main Findings from the original study	Main Findings from the replication study
Task shifting of antiretroviral treatment from doctors to primary-care nurses in South Africa (STRETCH): a pragmatic parallel, cluster-randomised trial by Fairall and colleagues (2012) [17]	1. Cox proportional hazards (PH) models and Huber-White robust adjustment of errors for intracluster correlation of outcomes	1. The Schoenfeld residuals test and cumulative sums of martingale-based residuals methods	The task-shifting program was not inferior to standard care: overall, no outcomes were worse in the task shifting intervention groups than in the standard care groups.	1. Pure replication validates the original findings.
				2. The MEA also validates the original findings:
				A. Overall, time to death did not differ between intervention and control patients;
		2. The generalized estimating equation (GEE) approach		
		3. The frailty model		
		4. The generalized linear mixed-effects model (GLMM)		
	2. Binomial regression			B. Rates of viral suppression, a year after enrolment, were equivalent in the intervention and control groups.
Effect of a cash transfer programme for schooling on prevalence of HIV and herpes simplex type 2 in Malawi: a cluster randomised trial by Sarah Baird and others (2012) [18].	1. An intent to treat analysis, using unadjusted and adjusted ORs with logistic regression models, with robust standard errors, which allows for intraclass correlation.	1. GLMMs (also known as hierarchical or multilevel models)	The intervention lowered the odds of HIV and HSV-2 prevalence in baseline schoolgirls but did not have a significant effect for baseline dropouts.	1. Pure replication validates the original findings.
		2. Permutation test		2. In the MEA, it was found that the intervention effect on HIV prevalence was somewhat sensitive to model choice.
		3. Bivariate outcome estimation		
				3. In the permutation analysis and in a GLMM, the intervention effect on HIV prevalence was no longer significant, but the results for HSV-2 prevalence were retained.
	2. Adjusted ORs were calculated, including age group and geographical stratum as fixed effects, as well as the baseline values for any behavioural outcomes.			
HIV development assistance and adult mortality in Africa [20]	1. A logistic regression and a difference-in-difference analysis to evaluate the effects of PEPFAR implementation. Specifically, the original authors compared the odds of adult (defined as men and women aged 15 to 59 years) all-cause mortality in focus and non-focus countries pre- and post-PEPFAR implementation.	1. The use of a logistic regression with the difference-in-difference model but limited to only observations between 2000 and 2006 (the Duber and colleagues (2010) time frame).	Between 2004 and 2008, all-cause adult mortality declined more in countries that implemented PEPFAR than countries that did not implement PEPFAR.	1. Pure replication validates the original findings.
				2. Bendavid and colleagues’ findings were robust to the time period used by Duber and colleagues (2010).
		2. A sensitivity analysis: a logistic regression on the unadjusted and adjusted model, leaving out any one country at a time, including only countries that had all data for 2000 to 2006, and using a linear time trend, as opposed to a dichotomous indicator, for PEPFAR implementation.		
				3. These findings did not match the findings from Duber and colleagues (2010) who find that PEPFAR may have little or no impact on health outcomes not explicitly targeted.
Timing of antiretroviral therapy for HIV-1 infection and tuberculosis by Havlir and colleagues (2011)[22]	The Pearson chi-square test to compare rates and the Kaplan–Meier method to produce unadjusted survival curves between the two study arms.	1. Adjustment for loss to follow-up	Earlier ART initiation (within two weeks of the initiation of treatment for TB) reduces the rate of new AIDS-defining illness and death exclusively in persons with CD4 counts lower than 50, as compared with later ART initiation.	1. Pure replication validates the original findings.
		2. An analysis of covariance (ANCOVA) specification, which consists of including the lagged outcome variable in the model specification to estimate the impact of the intervention.		
				2. Adjusting for loss to follow-up does not affect the main results of the paper.
				3. The use of an ANCOVA specification and instrumental variables weakened the main results. Specifically, the estimates from instrumental variables show that earlier ART initiation has no effect on the rate of new AIDS-defining illness and death for HIV positive TB patients with a CD4 count lower than 50. Thus, the MEA shows that the primary result of the paper may not be robust.
		3. The estimates in the original paper are from intention to treat, we use an instrumental variables approach to estimate the treatment effect on the treated		
The Regai Dzive Shiri Project: results of a randomised trial of an HIV prevention intervention for Zimbabwean youth by Cowan and colleagues (2011) [23]	1. The original paper conducted separate intent-to-treat analysis for males and females.	1. Evaluate the representativeness of the participants based on their characteristics	Despite an impact on knowledge, on some attitudes and on reported pregnancy, there was no impact of this intervention on HIV or HSV-2 prevalence, further evidence that behavioural interventions alone are unlikely to be sufficient to reverse the HIV epidemic.	1. Pure replication validates the original findings.
		2. Apply multilevel modelling to account for the hierarchical structure of the data		2. The amount of exposure to the intervention an individual received affected knowledge and attitude outcomes and a few risky sexual behaviours. However, increased knowledge and attitudes was not associated with decreased HIV or HSV-2 prevalence.
		3. Evaluate the impact of the intervention on multilevel attitude or knowledge outcomes		
		4. Evaluate the impact of the intervention among participants based on the level of the intervention they actually received		
	2. The unadjusted odds ratios (UORs) and AORs were computed using generalized estimating equations (GEEs) with exchangeable correlation and robust standard errors, which allowed for intraclass correlation among clusters.			
		5. Evaluate heterogeneous impacts of the intervention on HIV or HSV-2 among different age or history of risky sexual behaviour groups		
	3. When calculating the AORs, the GEE model included age, strata, marriage and highest level of education as fixed effects.			

Source: Authors ‘construction.

Chen and Alam’s replication study [10] re-examines Fairall and colleagues’ work on task shifting [17]. The original authors assessed the effects of task shifting, from doctors to primary care nurses in South Africa on several health and quality indicators. The study provided support for expanding the pool of ART prescribers beyond doctors to nurses, thus increasing access to ART, both by increasing the number of prescribers as well as the distribution to more remote areas.

Chen and Alam’s replication validates the original findings.

Smith, Hein, and Bagenda’ replication study [13] re-examines Baird et al.’s research on cash transfers and HIV/HSV-2 prevalence [18]. Baird et al.’s article is influential partly because it was among the first to question the impact of structural interventions, in this case, the cash transfer for schooling on HIV acquisition among young women. Overall, the study showed that cash transfers to unmarried schoolgirls could help reduce risky sexual activities and the likelihood that young women will be infected with HIV and HSV-2.

Smith, Hein, and Bagenda were able, generally, to replicate the original study. A theory of change analysis confirmed the causal pathway proposed in the original study—largely via school enrolment and selected sexual behaviors. In addition, they used the measurement and estimation analysis to confirm a criticism made by Webb and colleagues’ [19]. They showed the original results are sensitive to the methods used. The replication authors note that the low incidence of HIV made the results especially sensitive to model choice.

Hein, Bagenda and Jiangtao’s replication study [12] re-examines Bendavid et al.’s paper on HIV development assistance and adult mortality in Africa [20], based on data from The US President’s Emergency Plan for AIDS Relief (PEPFAR). PEPFAR is the largest initiative ever devoted to a single disease. Bendavid and colleagues found that between 2004 and 2008, all-cause adult mortality declined more in PEPFAR focus countries relative to non-focus countries [20]. By contrast, another study by Duber and colleagues [21] used a shorter timeframe and assessed varying groups of countries, but found no affect.

Hein, Bagenda and Jiangtao generally were able to replicate the original results. By limiting the data to only observations between 2000 and 2006 (inclusive) and testing different groups of countries, as Duber and colleagues did, [21], the replication authors find that Baird and colleagues’ results are robust to the different analyses.

Djimeu and Heard’s replication study [14] re-examines Havlir and colleagues’ paper [22] on the timing of concurrent HIV and TB treatment. Before 2011, initiation of ART was often deferred until completion of the intensive phase of TB therapy because of concerns about potential drug interactions, overlapping side effects, a high pill burden and programmatic challenges. Havlir and colleagues found that earlier ART initiation improves outcomes only for patients with a CD4 count less than 50 [22], suggesting that very sick patients could benefit by starting antiretroviral therapy earlier.

Djimeu and Heard’s were generally able to reproduce the original results. Although there were two major differences related to significance levels, neither difference affected the main findings or interpretation of the original study. The measurement and estimation analyses weakened the main findings from the original paper. Djimeu and Heard suggest that the choice of when to start ART should be based mostly on factors other than CD4 count similar to new evidence examining the optional timing for ART initiation, which suggest that earlier initiation has no effect even for patients with lower CD4 levels.

Finally, Yu, Hein, and Bagenda’s replication study [11] re-examines a paper by Cowan and colleagues on the effect of a multicomponent HIV prevention program among young people on HIV and HSV-2 acquisition, through improving knowledge and attitudes [23]. The paper is a pioneer in behavioural HIV prevention in that it assessed the effectiveness of the intervention on HIV prevention based on objective biomedical endpoints (prevalence). Previous studies have mostly assessed the impact of behavioural HIV prevention on knowledge, attitude, and, to some extent, behaviours. The aim of the study by Cowen and colleagues was to see whether changes in knowledge, attitude, and behaviours could lead to a reduction of HIV and HSV-2 acquisition. They found an impact of the intervention on prevalence of HIV or HSV-2 or current pregnancy. Yu, Hein and Bagenda’s replication study largely reproduced the original paper findings although some statistical codes were missing.

Cowan and colleagues noted a substantial amount of migration among participants. In the measurement and estimation analyses, Yu, Hein and Bagenda find a few substantial differences between newcomers and long-time residents that might have affected the final effects of the intervention. They also find that if the treatment group had been living in the community for the entire five years and was fully exposed to the intervention, that the treatment might have been more effective. Different model specifications did not substantially affect the results. The replication study rules out of the absence of an intrinsic lack of effect of the intervention *per se* and suggest that a better design of a similar intervention using lessons learnt from the original study might produce different results. However, a theory of change analysis showed that changes in knowledge and attitudes did not have a significant effect on HIV or HSV-2 prevalence.

## Learning and implications

The combination of these five studies provides several key insights. When sufficient data and code were available, the replication authors were generally able to conduct a PBR and reproduce the results published in the original papers with only minor differences. Any differences were due mostly to coding or typographical errors. In only one out of four PBRs, the replication researchers were not able to complete the PBR due to missing statistical codes for few original results. Similarly, pure replications using the original, described methods and data generally produced the same results as presented in the original paper. Overall, we find that we can generally trust that these, and likely most, papers can be replicated and, barring fraud, the results are as reported. It does however, highlight the need for authors to carefully double-check and copyedit their work to avoid typographical errors, and that they include all the codes needed to reproduce their results when they make their data and codes publicly available.

These replication studies also highlight the usefulness of reanalysing data with alternative estimation strategies. Two points emerge from using alternative estimation strategies. Studies that rely on relatively small pilots, that have few observations or clusters or rare and few outcomes of interest (e.g. less than 30 per study arm), should be externally replicated (repeated)—either in a different location or at larger scale, before being used to inform national or international policy. These results are not robust to alternative estimation models and therefore the effect may not be generalizable. For example, in Smith, Hein, and Bagenda’s replication study the original authors’ result for HIV prevalence relied on 24 observations (7 in the intervention group and 17 in the control group) and the results were weakened by alternative methods. The same observation can be made for the Havlir et al. study. The finding relied on 54 observations (17 in the intervention group and 38 in the control group). The results of alternative econometric methodologies do not support the main findings from the original paper. While we do not have enough evidence or data to claim that the weakness of the original results was due to the small sample size, these replications nevertheless highlights the fact that results obtained with a very small sample size, or a rare outcome with few observations, might not be robust. Thus, any formulation of policy recommendation from such studies should be scrutinized using alternative methods, or by conducting another similar trial with a bigger sample to ensure policy recommendation are based on reliable and replicable evidence.

Furthermore, the replication studies presented in this collection show that replication offers an opportunity to fill some gaps, such as helping to explain why the intervention evaluated worked or not. As most scientific journals impose a word count restriction, many studies, including those replicated in this collection, only present the intervention and the results and do not seek to understand/explain the results found other than through theory or supposition.

The theory of change analysis in the replication studies provides this opportunity. Thus Smith, Hein, and Bagenda are able to show that the effect of the intervention (cash transfers) is obtained mainly through the school enrolment and to a lesser extent through having unprotected sexual intercourse. Additionally, Yu, Hein, and Bagenda show that the lack of reduction of HIV or HSV-2, despite improvements in knowledge and attitudes due to the multicomponent prevention intervention, might be due to low exposure to the intervention and not the intervention itself. They show that differential levels of exposure might have different impacts on both the knowledge and attitude and on HIV and HSV-2 prevalence. Their results suggest that we should reassess the intervention while ensuring proper implementation and target population exposure before concluding whether the intervention is effective.

In short and especially related to the theory of change assessment, a theory of change clearly lays out how and why one could expect the intervention to improve outcomes. Improving health outcomes will only occur if the intervention is both effective and lies along the causal pathway. While more studies should include both a theory of change and an assessment of whether it holds, replication studies can fill gaps when word counts limit what primary analyses can publish.

Replication studies are a public good that provide verification of whether results are robust and can provide additional insights into how and why interventions might work. These additional analyses are important to ensure policy formulation is based on robust and correctly explained results and do not dismiss failed interventions without knowing whether it was a failure of implementation or a failure of the intervention. Finally, these replication studies demonstrate the importance of replicating smaller pilots or studies with rare and few outcomes of interest before adopting policy recommendations based on their results.

## Conclusion

This paper defines different forms of replication and demonstrates the value of not only performing the replication studies but also publishing them in a peer reviewed journal. We introduce the replication studies, included in this collection, of five influential studies on biomedical, social, behavioural and structural interventions for HIV prevention and treatment. Our overview shows the authors were generally able to reproduce the results published in the original papers through PBR and pure replication. However, we also find that when results are from a small sample size or rare outcome, results are not robust to alternative models. Furthermore, the theory of change analyses fill gaps in explaining results and highlight the fact that this additional analysis should be encouraged. It allows a better understanding of why an intervention works or does not and helps determine whether, in cases of no effect, whether the failure is a failure of implementation and should be repeated or a failure of the intervention. Finally, replication studies, both confirmatory and contradictory, increase confidence that influential studies are robust and can be relied on for policy.

Publishing these studies has not always been a priority for top journals. Some reviewers argue that these studies are not innovative and do not contribute new knowledge. We argue that these studies are both valuable and that they make a significant contribution to the literature. More journals need to accept and publish these important studies. Without publication, their results, and the value provided of verifying or modifying or explaining conclusions, is undermined or eliminated.
